# Estimation of biological effect of Cu-64 radiopharmaceuticals with Geant4-DNA simulation

**DOI:** 10.1038/s41598-022-13096-2

**Published:** 2022-05-27

**Authors:** Tamon Kusumoto, Kentaro Baba, Sumitaka Hasegawa, Quentin Raffy, Satoshi Kodaira

**Affiliations:** 1 National Institutes for Quantum Science and Technology, 4-9-1 Anagawa, Inage-ku, Chiba, 263-8555 Japan; 2 Graduate School of Biomedical Science and Engineering, Hokkaido University, Kita-15 Nishi-7, Kita-ku, Sapporo-shi, Hokkaido, Japan; 3 Institut Pluridisiplinaire Hubert Curien, UMR 7178, CNRS, 23 rue du Loess, Strasbourg, France

**Keywords:** Biophysics, Medical research

## Abstract

The aim of this work is to estimate the biological effect of targeted radionuclide therapy using Cu-64, which is a well-known Auger electron emitter. To do so, we evaluate the absorbed dose of emitted particles from Cu-64 using the Geant4-DNA Monte Carlo simulation toolkit. The contribution of beta particles to the absorbed dose is higher than that of Auger electrons. The simulation result agrees with experimental ones evaluated using coumarin-3-carboxylic acid chemical dosimeter. The simulation result is also in good agreement with previous ones obtained using fluorescent nuclear track detector. From the results of present simulation (i.e., absorbed dose estimation) and previous biological experiments using two cell lines (i.e., evaluation of survival curves), we have estimated the relative biological effectiveness (RBE) of Cu-64 emitted particles on CHO wild-type cells and xrs5 cells. The RBE of xrs5 cells exposed to Cu-64 is almost equivalent to that with gamma rays and protons and C ions. This result indicates that the radiosensitivity of xrs5 cells is independent of LET. In comparison to this, the RBE on CHO wild-type cells exposed to Cu-64 is significantly higher than gamma rays and almost equivalent to that irradiated with C ions with a linear energy transfer of 70 keV/μm.

## Introduction

It is known that radiotherapy for cancer is a treatment that allows maintaining a good Quality of Life (QOL) of patients. Conventional external radiotherapy with X-ray therapy and particle therapies (e.g., proton and carbon ion therapy) are appropriate for the treatment of solid cancers^1^. In comparison, targeted radionuclide therapy (TRT) is effective for eradicating disseminated cancers^2^. In TRT, the effect of the use of α emitters (e.g., At-211 and Ra-223) and β emitters (e.g., Y-90 and I-131) has been demonstrated through radiobiological experiments with mice and living cells e.g.,^3–6^. Some β emitters are utilized in treatment protocols, for instance, Y-90 on non-Hodgkyn’s lymphoma (NHL)^7^ and metastatic prostate cancer and I-131 on thyroid and NHL^8,9^). In addition, applicability of Auger electron emitters (e.g., Cu-64, Ge-71, Pt-193 m and In-111) to TRT has been investigated by many authors e.g.,^10–12^. Auger electrons have high Linear Energy Transfer (LET) compared to β rays and shorter range than both α particles and β rays. Therefore, the applicability of Auger electron emitters to TRT is actively being discussed.

Radiolabeled copper (II) (diacetyl-bis N4-methylthiosemicarbazone) (Cu-ATSM) preferentially accumulates in hypoxic tumors^13^. Therefore, among Auger electron emitters, Cu-64 is receiving a lot of attention for the efficient treatment of hypoxic tumors. TRT using Cu-64 was comprehensively reviewed^14–20^. Cu-64 decays by emission of beta particles (i.e., β rays with 0.573 MeV energy in 40% cases, positrons with 0.656 MeV energy in 19% cases) and electron capture in 41% cases^21^. Electron capture results in cascades of Auger electrons^22^. Since Cu-64 is not only an Auger electron emitter but also a positron emitter, it allows to diagnose and therapize cancers at the same time, that is performing “theranostics” (coined word of therapeutics + diagnostics). This point of view also highlights the benefit of use of Cu-64 for TRT. In comparison to this, the range of beta particles is much longer than the size of cell, meaning that healthy tissues surrounding tumors could be affected by beta particles. Thus, the influence of beta particles emitted by Cu-64 should be clarified to minimize the medical exposure. For a reliable treatment using Cu-64, not only the therapeutic effect but also the risk of medical exposure should be quantitatively estimated. However, direct measurements of the absorbed dose by Auger electrons were not performed, meaning that the effect of TRT using Auger electrons could not be directly compared with that of other radiation therapies. It is considered that the dose assessment is crucial for the reliable treatment using Cu-64.

Previously, we have demonstrated the possibility to measure dosimetry signals due to Auger electrons emitted from Cu-64 source using Fluorescent Nuclear Track Detector (FNTD)^23^, a single aluminum oxide crystal doped with carbon and magnesium (Al_2_O_3_: C,Mg), engineered by Landauer^24^. The mean range of Auger electrons in water is about 120 nm, which is much smaller than that of beta particles emitted by Cu-64. Thus, by acquiring the depth dependence of the fluorescence intensity by confocal microscopy, we successfully discriminated the signal of Auger electrons from that of beta particles. We estimated that the absorbed dose of Auger electrons is almost equivalent to that of beta particles at 1 μm depth from the Cu-64 source^23^, where the contribution of Auger electrons is significant (i.e., Auger electrons deposited their energies from surface to 1 μm depth of FNTD). Furthermore, simulations were done to estimate the absorbed dose of Cu-64^25^. However, the biological effectiveness of TRT using Auger electron emitters was not discussed. In the present study, we have simulated the absorbed dose of particles emitted by Cu-64 using the Geant4-DNA Monte Carlo simulation code in the cell level. The obtained simulation results are experimentally validated using coumarin-3-carboxylic acid (C3CA) solution, a well-known scavenger of hydroxyl radicals e.g.,^26,27^, which efficiently react with DNA and proteins^28,29^, and can be used as a chemical dosimeter^30^. Then, biological effectiveness was discussed based on the absorbed dose calculated by the Geant4-DNA simulation and surviving fraction of cells exposed to Cu-64.

## Materials and methods

The Geant4-DNA Monte Carlo simulation code ver. 10.05.p01 was used to calculate the absorbed dose of particles emitted by Cu-64^31–33^. In the present simulation, we calculated the absorbed dose of all particles produced by the decay in Cu-64 (i.e., beta particles, Auger electrons and characteristic X-rays) in the simulation world, whose geometry was air-free water, in a spherical shape. The “G4RadioactiveDecay” class and “G4EmStandardPhysics_option4” physics constructor were used to simulate the radioactive decay. To take into account the influence of low-energy secondary electrons, their deposited energies were calculated using the “G4EmDNAPhysics_option2”, in which the discrete processes including ionization, electronic excitation, vibrational excitation, elastic scattering and molecular attachment were followed for energies down to 7.4 eV^34^. We followed all electrons produced down to 7.4 eV. When energies of electrons produced are lower than 7.4 eV, they deposit their all energies at generated locations. The Cu-64 sources were randomly distributed in the water sphere. To investigate the contribution of Auger electrons, we calculated the absorbed dose of Cu-64 both with and without Auger electron emission. The size of the sphere was varied from 10^–11^ to 10^3^ μL (from 1.3 × 10^–4^ to 6.2 mm in radius), while maintaining the radioactivity of Cu-64 per volume. In the simulation, we set the radioactivity of Cu-64 as 1 Bq/μL. The number of total decays during the simulation was 10^5^.

From the absorbed dose assessed by the Geant4-DNA simulation and previously evaluated survival curves using two cell lines (CHO wild-type cell and xrs5 cell)^10^, we estimated the relative biological effectiveness (RBE). The values of RBE estimated are compared to those of gamma rays, protons and heavy ions to discuss the effectiveness of TRT using Auger electron emitter.

We purchased radiation labeled aqueous copper chloride solution from Fujifilm/Wako Pure Chemical Industries Ltd. For the irradiation with Cu-64 solution, C3CA (purity > 98%; Fujifilm/Wako Pure Chemical Industries Ltd., Japan) solutions were prepared from ultrapure water (Milli-Q Advantage; Merck&Co., U.S.A.). The molar concentration of C3CA solution was 0.5 mM. Additionally, 0.5 mM C3CA solution was prepared in 1/15 M phosphate buffer, pH 6.8 (Fujifilm/Wako Pure Chemical Industries Ltd., Japan) for gamma exposures. The C3CA solution was pure enough for quantitatively evaluating the amount of hydroxyl radicals produced.

Under irradiation, C3CA yields a highly fluorescent compound, 7-hydroxy-coumarin-3-carboxylic acid (7OH-C3CA) upon reaction with hydroxyl radicals produced by water radiolysis^26^. The 7OH-C3CA produced was separated from other products (e.g., C3CA) by HPLC (Prominence-2200, SHIMADZU, Japan), using Hypersil Gold C18 column (250 × 4.6 mm, i.d. 5 μm) at a flow rate of 0.8 mL/min at 25℃. Fluorescence was measured with a fluorescence detector (RF-20A, SHIMADZU, Japan), with an excitation wavelength of 370 nm by a Xe lamp and an emission wavelength of 410 nm. Amount of 7OH-C3CA produced after irradiation, was determined using the analytical curve reported previously^35^. The yield of formation of 7OH-C3CA with hydroxyl radicals has been determined^27^, therefore its concentration gives to access the radical yield.

Aqueous copper chloride solution (0.05 M), labeled by Cu-64, was added to 0.5 mM C3CA solutions contained in 200 μL PCR tubes. The radioactivity of Cu-64 was 0.2 MBq/μL. At first, we prepared C3CA solutions made by ultrapure water with 9, 18, 27, 36, 45 and 54 μL. Then, 1 ,2, 3, 4, 5 and 6 μL of copper chloride solutions were added to the C3CA solutions, to reach 10, 20, 30, 40 50 and 60 μL final volumes, respectively. The exposure duration was 24 h for all solutions. Namely, we followed the volume dependence of 7OH-C3CA formed.

**Figure 1:**
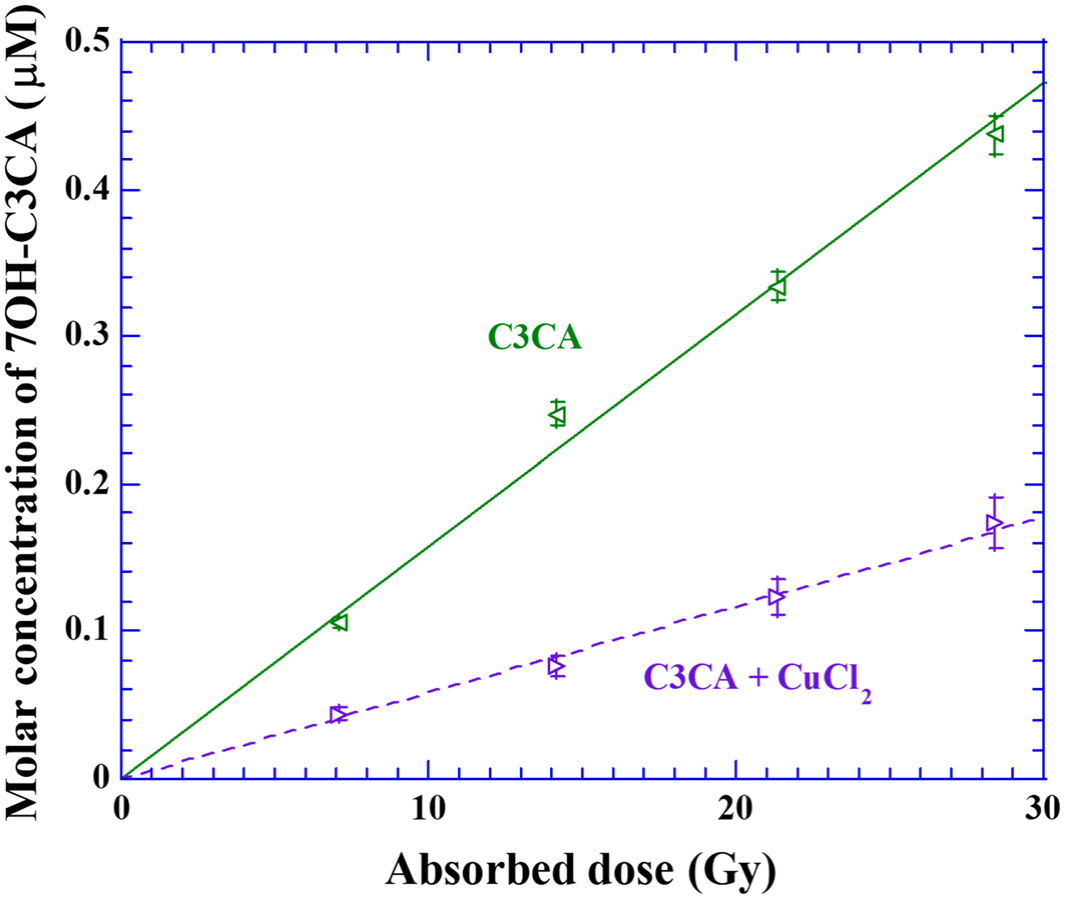
Molecular concentration of 7OH-C3CA formed after exposures to gamma rays from Cs-137. The leftward triangles represent the molar concentration of 7OH-C3CA formed in C3CA solution made by 1/15 M phosphate buffer and the rightward triangles show that made by ultra pure water with non-radioactive copper chloride solution.

The copper chloride solution is acidic, so that the scavenging process of hydroxyl radicals by C3CA would be affected by the modification of pH. Furthermore, the chlorine acts as a radical scavenger with a rate constant k = 3.1 × 10^9^ M^-1^ s^-1^. Namely, the scavenging process of hydroxyl radicals by C3CA could compete with chlorine. So, prepared C3CA solutions were exposed to gamma rays from Cs-137 with and without addition of non-radioactive copper chloride solutions to assess its chemical effect on the hydroxyl radical scavenging, whether by competition with C3CA or by change in the pH (Fig. 1) in the National Institutes for Quantum Science and Technology (QST)/ National Institute of Radiological Sciences (NIRS). The gamma irradiations were performed with a dose rate of 6.2 Gy/min. The volume of solutions exposed was 2 mL. The molar concentration of 7OH-C3CA was determined by HPLC-fluorescence. It increases monotonically with increasing absorbed dose in both cases. The molar concentration of 7OH-C3CA in the C3CA solution in phosphate buffer rises more sharply with dose than that with copper chloride solution. The difference between the slope integrates both pH effect and the influence of scavenging hydroxyl radicals by chlorine in C3CA solution. To correct the influence of copper chloride solution, a correction factor of 2.6 is used, obtained from the ratio between the slopes.

## Results and discussion

### Dose estimation of Cu-64 by Geant4-DNA

**Figure 2:**
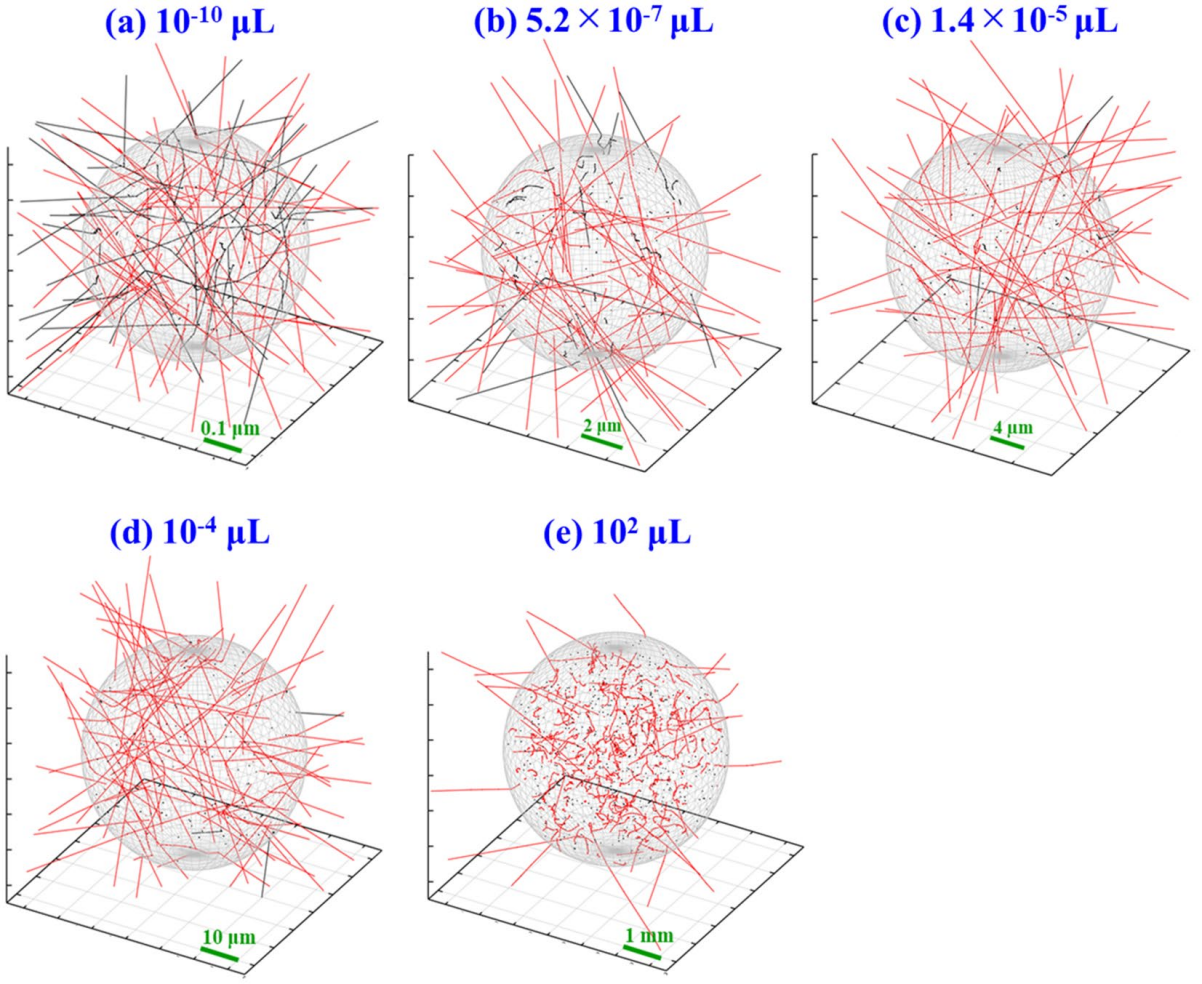
Simulated trajectories of Auger electrons (black) and beta particles (red) in water spheres with (**a**) 10^–10^, (**b**) 5.2 × 10^–7^ (**c**) 1.4 × 10^–5^ (**d**) 10^–4^ and (**e**)10^2^ μL. The trajectories of secondary particles were not described. The sizes of spheres with 5.2 × 10^–7^ and 1.4 × 10^–5^ μL are concordant with radii of 5 and 15 μm, respectively, and correspond to the size of cell nucleus and cytoplasm.

Auger electrons from Cu-64 have an average energy of 2 keV, whose range is about 120 nm in water^37^. In comparison to this, the continuous slowing down approximation (CSDA) range of electrons with 0.573 MeV is 2.1 mm, which is six orders magnitude longer than that of Auger electrons^38^. Figure 2 illustrates trajectories of Auger electrons (black) and beta particles (red) emitted from Cu-64 sources randomly distributed in the water sphere. Here, we do not describe the trajectories of secondary particles. The volumes of water sphere are (a) 10^–10^, (b) 5.2 × 10^–7^ (c) 1.4 × 10^–5^ (d) 10^–4^ and (e) 10^2^ μL. We note here that the sizes of spheres with 5.2 × 10^–7^ and 1.4 × 10^–5^ μL are concordant with radii of 5 and 15 μm, respectively, and correspond to the size of cell nucleus and cytoplasm. We illustrate these pictures with the number of decays with 250. In the case of the 10^–10^ μL sphere, Auger electrons deposit their energies not only inside the sphere but also outside from it. A similar trend is seen in beta particles in this volume. At 5.2 × 10^–7^, 1.4 × 10^–5^ μL and 10^–4^ μL, almost all Auger electrons terminate their trajectories in the water sphere, while beta particles still deposit their energies outside the water sphere. When the volume of water sphere increases further, for example at 10^2^ μL, only a few beta particles come out of the water sphere, for example at 10^2^ μL.

**Figure 3:**
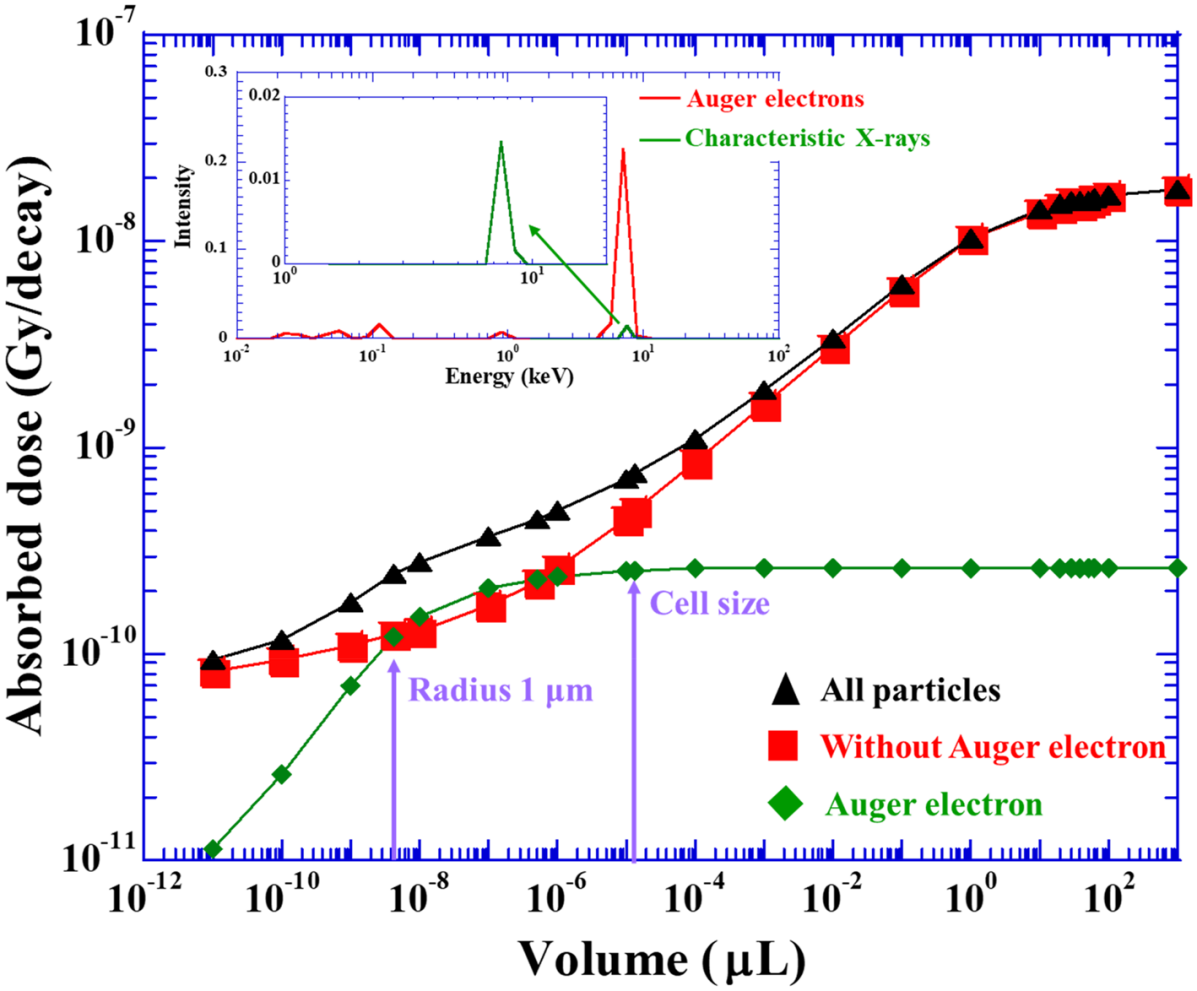
The absorbed dose of emitted particles from Cu-64 source as a function of the volume of water sphere. The triangles represent the results of full simulation. The result of simulation without the contribution of Auger electrons is also shown as the squares. The diamonds express the influence of Auger electrons. Error bars are smaller than the size of plot, therefore, they are not visible. Arrows indicate the absorbed dose at the volume with radius of 1 and 15 μm. The latter is equivalent to the average size of cells. The inset is energy spectra of Auger electrons (red) and characteristic X-rays (green).

From the absorbed dose and the number of decays during the simulation, we evaluate the absorbed dose per decays as a function of the volume of water sphere (Fig. 3). The inset graph is an energy spectrum of Auger electrons considered in the present work for the calculation of the absorbed dose. Overall, the absorbed dose simulated with Geant4-DNA increases monotonically with increasing the volume of water sphere. In this simulation, we evaluate the absorbed dose of the radiations emitted by Cu-64 with (triangles) and without contribution of Auger electrons (open squares). The absorbed dose from Auger electrons only is also evaluated (diamonds).

Auger electrons are launched in 22% of total decays. Since the number of Auger electrons emitted (i.e., fluence) is lower than that of beta particles, the absorbed dose of Auger electrons is smaller than that of beta particles below 10^–10^ μL, in which not only beta particles but also Auger electrons deposit their energies outside of the sphere. With increasing volume of the water sphere, the contribution ratio of Auger electrons to the absorbed dose increases due to their higher LET. The absorbed dose of Auger electrons becomes constant above 10^–5^ μL. Notably here that, the peak energy of characteristic X-rays of Cu-64 is 7.5 keV, whose attenuation length is about 800 μm (inset of Fig. 3; the detail ratio of X-rays is l (0.85 keV): 0.5%, kα1 (7.48 keV): 9.4%, kα2 (7.46 keV): 4.8%, kβ1 (8.27 keV): 1.1%, kβ3 (8.27 keV): 0.58%). Thus, secondary electrons produced by characteristic X-rays of Cu-64 deposit most of their energies outside of the water sphere, resulting in the low contribution to the absorbed dose relative to beta particles and Auger electrons (not plotted). Considering a sphere of 1 μm radius (i.e., 4.2 × 10^–9^ μL, shown by arrow in Fig. 3), the absorbed dose of Auger electrons is almost equivalent to that of beta particles. This finding supports our previous results measured with FNTD^23^. The absorbed dose increases monotonically with the volume of the water sphere. Above 10 μL, the increasing trend slows down. This result shows that the proportion of beta particles that end their trajectories in the water sphere becomes significant. Above 10^2^ μL, the absorbed dose is constant with the volume of the water sphere, resulting in not only Auger electrons but also beta particles terminate their trajectories in water sphere.

### Validation of Geant4-DNA simulation with measurements

**Figure 4:**
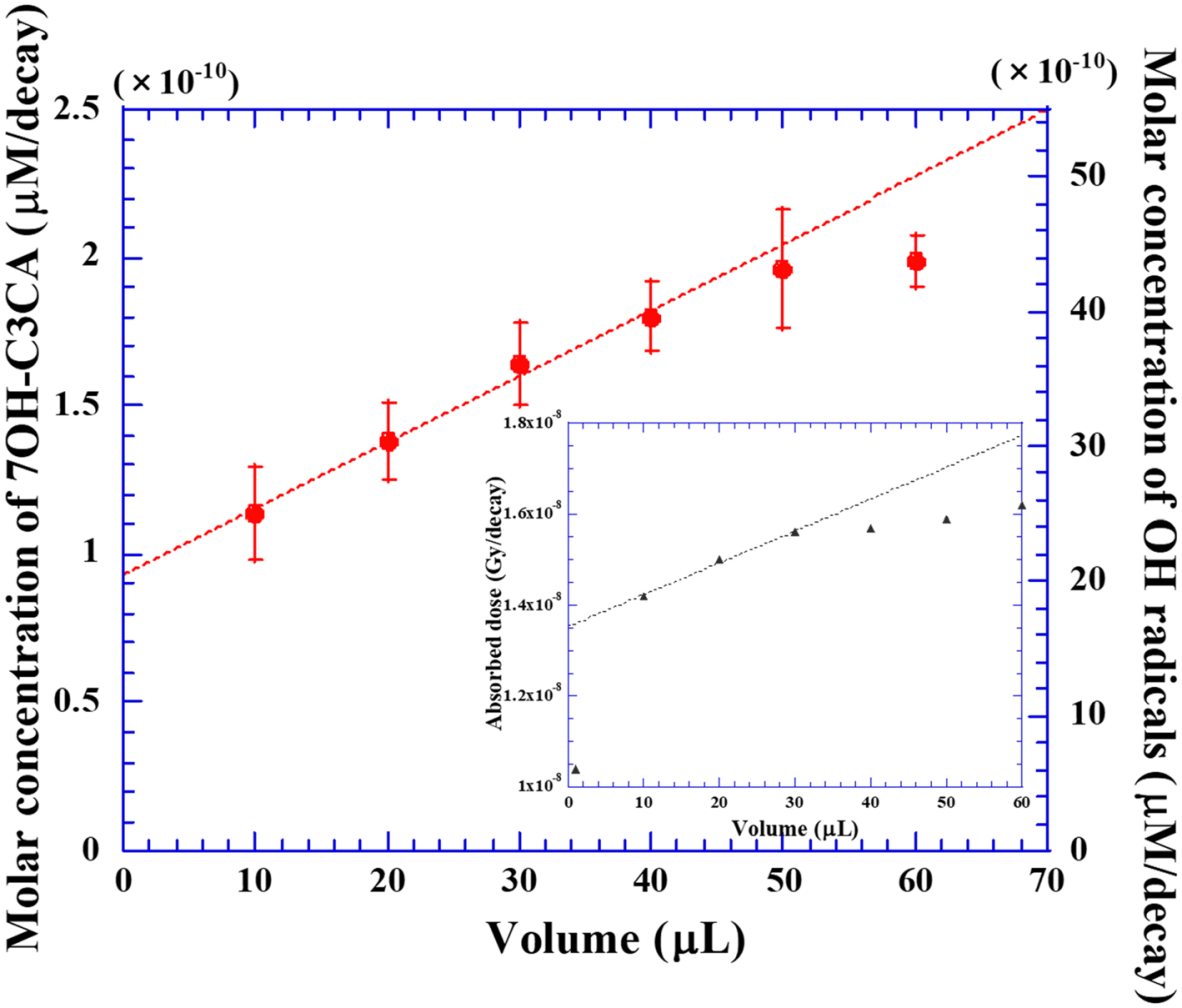
Relation between molecular concentration of 7OH-C3CA formed after exposures to Cu-64 (left axis) and the volume of solution. Molar concentration of hydroxyl radicals against the volume of solution is also represented (right axis). The inset graph is a simulation result enlarged from 10 to 60 μL. The dashed lines in graphs are results of fitting using a linear function (y = ax + b).

To allow comparison with simulation, solutions of increasing volumes and identical radiation labelled copper salt concentration are prepared. The amount of hydroxyl radicals per disintegration is measured to assess the influence of total volume, in which the chemical dose is deposited on the radical yield. Figure 4 represents the concentration of 7OH-C3CA formed per one decay (left axis) of Cu-64 as a function of the volume of solution. The concentration of 7OH-C3CA can be derived from the calibration curve as previously reported^36^. It is recognized that 4.7 ± 0.6% of hydroxyl radicals are converted to 7OH-C3CA independently not only on radiations but also on LET, therefore, the number of hydroxyl radicals is also represented (right axis). The dashed lines are fitting results by a linear function (y = ax + b), made in the linear portion of the data, below 30 μL. At 0 μL, the absorbed dose should be 0. However, the fitting curve obviously has a y-intercept (Fig. 4). Since the range of beta particles is about three orders of magnitude longer than that of Auger electrons, the contribution of Auger electrons might appear on the intercept. Turning our attention to the simulation (please see the inset graph of Fig. 4: Simulation results enlarged from 0 to 60 μL in linear scale), the increasing trend of the absorbed dose is slowing down above 10 μL (Fig. 3), resulting in the finding of a y-intercept as seen in the experimental result.

To compare the effect of various modalities of irradiation, one needs some common parameter. In a case like Auger electrons, where the spatial distribution of the dose absorption plays a critical role, the physical dose in Gy is not the best parameter to allow comparison of the effect with other radiations. A chemical dose, the quantity of hydroxyl radicals formed in water by radiolysis with an Auger emitter, would allow more accurate comparison, taking into account the first step of the chemical effect of radiolysis. This is what is proposed here, using C3CA as a chemical probe to estimate the amount of hydroxyl radicals produced by water radiolysis.

Radiation chemical yield (G value), represents the number of entities formed or destroyed by unit energy (usually 100 eV) deposited in the medium. By measuring the quantities of 7OH-C3CA formed, the G value of hydroxyl radicals can be determined, as the yield of the reaction of formation of 7OH-C3CA is known^26^. Namely, C3CA solution acts as a chemical dosimeter^30^, which applicability was demonstrated in a thermal neutron field^36,40^. The number of hydroxyl radicals formed would become the standard for evaluating the biological effect induced by radiations^40^. When the G value of hydroxyl radicals (7OH-C3CA) is known, the absorbed dose of certain ionizing radiations can be evaluated from the number of 7OH-C3CA formed. The advantage of the use of C3CA solution as a dosimeter is that Co-60 equivalent dose, which is the reference radiation, can be evaluated in water with neutral pH. That means that we can evaluate the Co-60 equivalent dose from the product of the number of hydroxyl radicals formed under a certain ionizing radiation and the G value for gamma rays from Co-60.

The number of hydroxyl radicals (7OH-C3CA) formed is proportional to the absorbed dose. From the fitting line represented in Fig. 4, the concentration of hydroxyl radicals (7OH-C3CA) formed increases 1.3 ± 0.2 times from 10 to 30 μL. In comparison to this, the absorbed dose estimated with simulation rises 1.1 ± 0.1 times in the same range (see inset of Fig. 4). A possible discrepancy might come from the difference between the geometry, as in the simulation the geometry is considered perfectly spherical, while experiments were carried out in a PCR tube. Therefore, we consider that the experimental results and simulation agree within the experimental uncertainty and the limitations of the models used.

As mentioned above, it is possible to evaluate Co-60 equivalent dose from the combination of the number of hydroxyl radicals (or 7OH-C3CA) formed with the G value for gamma rays from Co-60 or Cs-137 sources. Notably here that the G value of hydroxyl radicals for gamma rays from Co-60 is almost equivalent to that from Cs-137^41^. The G value of hydroxyl radicals formed in C3CA solutions with 0.5 mM for gamma rays from Co-60 is 2.7 /100 eV^26^. From this G value and the number of hydroxyl radicals formed in the 30 μL volume of C3CA solution, the absorbed dose of emitted particles by Cu-64 is determined as 1.3 × 10^–8^ Gy/decay. The absorbed dose evaluated by the simulation is of the same order, but a bit higher, 1.6 × 10^–8^ Gy/decay. The G value decreases monotonically with decreasing photon energy down to about 0.5 keV and then increases^41,42^. Therefore, a lower “gamma rays-equivalent” dose is coherent with higher yield of hydroxyl radicals with Auger electrons and beta particles compared with Co-60 gamma rays. Therefore, it is reasonable that the “gamma rays-equivalent” dose is smaller than the simulation. Therefore, the simulation result is validated by the dose estimation using C3CA solution.

### Comparison of RBE determined for Cu-64 from the absorbed dose

Previously, we implied that the number of OH radicals produced could become a universal parameter for expressing the biological effect^43^. Thus, based on the number of OH radicals produced, we could universally express the biological effect under ionizing radiations with different radiation qualities^30^. The absorbed doses of emitted particles from Cu-64 can be evaluated by the Geant4-DNA simulation. The contribution of Auger electrons to the absorbed dose is fairly small compared to that of beta particles (Fig. 3). Despite the small contribution of Auger electrons to the absorbed dose, a high biological effect induced by Auger electron emitters has been reported^10^. This finding implied that low-energy electron (LEE) impacts played important roles to lethal effect of cell as discussed below. The mean tissue cell radius including cytoplasm is 15 μm (i.e., 1.4 × 10^–5^ μL) (see arrow in Fig. 2). We should note here that the range of Auger electrons is much shorter than that of the size of cells. Therefore, the distribution of Cu-64 source within the cell is important for considering the biological effect induced by Auger electrons. However, the distribution of Auger electron source within the cell is an open question for the moment. Below, we discuss the biological effect of Auger electrons based on the previous results using ATSM as a delivery agent^10^, which has a characteristic of the accumulation in hypoxic cancer, under the assumption of the homogeneous distribution of Cu-64 source in the cell.

When the Cu-64 source is homogeneously distributed in the tissue cell, the absorbed dose per decay is 7.7 × 10^–10^ Gy/decay. The radioactivity of Cu-64 per cell, at which 10% of cells survive under the ionizing radiation, of CHO wild-type and xrs5 represent 0.97 and 0.40 Bq/cell, respectively^10^. The irradiation duration, from the uptake to the washing, is 5 h, so the number of decays can be simply calculated, resulting in the determination of the absorbed dose of emitted particles from Cu-64. Thus, we can evaluate D_10_ values, absorbed doses, at which 10% of cells survive under the ionizing radiation of particles emitted from Cu-64 from the present simulation (Table 1). Briefly, the CHO wild-type cells are more resistant to radiations than xrs5 cells^10^. The absorbed doses for reaching D_10_ are 2.23 and 0.92 Gy for CHO wild-type cells and xrs5 cells, respectively. These values are lower than previously reported values using MCF7/HER2-18 cells and LNCaP cells^44,45^ but in the same order magnitude. The discrepancy would come from the difference of the cell line. Here, Since the distribution of cells is sparse in vitro experiments compared to that in vivo experiments, the influence of the absorbed dose of beta particles from neighboring cells was not considered in our simulation.

**Table 1:** Values of D_10_ and RBE of various types of radiations^10^. LETs of gamma rays from Co-60, protons, C and Fe ions are also listed.

	LET (keV/μm)	CHO wild-type		xrs5	
		D_10_ (Gy)	RBE	D_10_ (Gy)	RBE
Gamma (Co-60)	0.3	6.37	1	1.18	1
Proton	1.1	5.31	1.20	1.16	1.02
C	13	3.79	1.68	0.91	1.30
	70	2.49	2.56	0.94	1.26
Fe	200	1.89	3.37	1.00	1.18
Cu-64	–	2.23	2.53	0.92	1.12

The RBE is defined as the survival at a given level (often 10% survival) for a certain radiation relative to that for gamma rays or X-rays^46^. The RBE values of CHO wild-type cells exposed to C and Fe heavy ions are higher than that of gamma rays from Co-60 (Table 1). In comparison to this, the RBE with xrs5 cells does not depend significantly on LET^10^. In the present study, RBE of Cu-64 is estimated from the D_10_ values evaluated with Geant4-DNA. The value of RBE of xrs5 cells exposed to Cu-64 is 1.12, which is almost equivalent to that of other radiations. In accordance with previous results, the surviving fraction of xrs5 cells decreased more rapidly with increasing absorbed dose compared to CHO wild type cells^10^. Additionally, extensively enough damage in xrs5 cells relative to that in CHO wild type cells was observed by the chromosomal aberrations assay^10^. Thus, the dependence of biological effect on the radiation quality is hardly seen due to the high radiosensitivity of xrs5 cells. In comparison to xrs5 cells, the RBE of CHO wild type cells exposed to radiations emitted from Cu-64 is 2.53. This value is higher than that of proton (LET: 1.1 keV/μm) and C ions (LET: 13 keV/μm) and is almost equivalent to that of C ions (LET: 70 keV/μm), so that TRT using Auger electrons can be an effective treatment for cancer. Simply speaking, high RBE value implies a lower absorbed dose required for cell killing, thereby the medical exposure by beta particles would be also lowered. This point of view also supports the benefit of TRT using Cu-64.

At the cell size, the contribution of beta particles to the absorbed dose is more than twice higher than that of Auger electrons. Nevertheless, the value of RBE of CHO wild type cells is higher than those of gamma rays and is equivalent to that of C ions (LET: 70 keV/μm). This finding implies that the absorbed dose could not be a universal parameter to describe the biological effect. Several models were developed to accurately express the biological effect based on the probability densities of domain and cell nucleus specific energies that successfully reproduced experimental data^47^. However, we must emphasize here that the higher RBE value expresses a high contribution of LEEs to the cell death as discussed in the section below.

The range in water of Auger electrons with mean energy of 2 keV is about 120 nm. In the case of Auger electrons, LETs increase drastically when their energy decreases (e.g., LET of 2 keV electron is 7 keV/μm and that of 150 eV is 26 keV/μm)^48^. The LET of Auger electrons is close to those of protons and C ions. However, the reactions induced by Auger electrons are far from those by energetic protons and C ions. Protons and C ions form cylindrical track cores due to their high ionization density associated with secondary electrons, of which energies are widely distributed^49^. Meanwhile, the density of emitted particles from Cu-64 would be lower than that of secondary electrons around the track core of proton or C ion track. So, discrete damage can be expected by beta particles and Auger electrons. In other words, the LET of Auger electrons should not be treated as the same classification as those of protons and C ions.

### Role played by LEEs (Hypothesis)

As mentioned above, we assume that the cell is an ideal sphere with a volume of 1.4 × 10^–5^ μL. At this volume, the contribution of Auger electrons to the absorbed dose is about half than that of beta particles. If the contribution of beta particles was dominant in cell killings, the RBE of CHO wild type cells exposed to Cu-64 source should be close to that irradiated with gamma rays. The high RBE value of Cu-64, comparable to C ions, would be explained by a significant contribution of LEEs at the end of trajectory of Auger electrons in cell killing. A significant increase in yields of DNA strand breaks was observed under irradiation by soft X-ray with phosphorus K-shell absorption energy, meaning that Auger electrons can efficiently damage DNA molecules^50^. Furthermore, it was clarified that clustered DNA damage, typically observed with high LET radiations, such as Si and Fe ions, were also induced by Cu-64 source^51,52^. Namely, clustered DNA damage could occur around the track end of electrons^53^. Auger electrons emitted by Cu-64 can ionize or excite a molecular unit of DNA around the beginning of trajectory. Then, Auger electrons gradually lose their energies and could cleave molecular unit of DNA at close locations of the ionized or excited site via dissociative electron attachment, which resonance energy is typically observed around 8.5 eV^54^, dissociative electron transfer (< 1 eV)^55^ and other LEE impacts (e.g., dipolar dissociation and dissociative ionization)^56^. Thus, “consecutive” damage to DNA molecule can be expected under exposures to Auger electrons. Such LEE impacts would largely contribute to clustered DNA damage leading to cell death. This fact indicates that it is not possible to universally describe the yield of DNA damage and surviving fraction of cells by LET and absorbed dose only. We should focus on the number of interactions with a molecular unit of DNA, including roles played by LEEs, to elucidate the mechanisms of cell killings^57,58^. The large RBE of Cu-64 demonstrates the effect of LEE impacts on DNA molecules.

## Conclusions

In this study, we evaluated the absorbed dose of emitted particles from Cu-64, which is an efficient Auger electron emitter, using a Monte Carlo simulation code using Geant4-DNA. The absorbed dose of Auger electrons in a water sphere with 4.2 × 10^–9^ μL was almost equivalent to that of beta particles. This result was in agreement with previous one obtained using FNTD. The absorbed dose by emitted particles from Cu-64 in a 30 μL water sphere was 1.6 × 10^–8^ Gy/decay, which agrees with Co-60 equivalent dose evaluated using C3CA chemical dosimeter. These findings demonstrated that the absorbed dose of emitted particles from Cu-64 was appropriately simulated using Geant4-DNA.

From the absorbed dose evaluated by Geant4-DNA, we estimated RBE of Cu-64 emitted particles towards CHO wild type and xrs5 cells. The RBE on xrs5 cells was found similar to the other particles, not much influenced by LET. This result could be induced by the high radiation sensitivity of xrs5 cells. In comparison to this, the RBE on CHO wild type cells exposed to Cu-64 was almost equivalent to that with C ions of LET of 70 keV/μm. At the cell size, the absorbed dose of beta particles was more than twice higher than that of Auger electrons. Nevertheless, the fact that the RBE of Cu-64 particles was much higher than that of gamma rays implied that LEEs played an important role in inducing clustered DNA damage leading to cell death.
